# Immunoproteasome Genes Are Modulated in CD34^+^ JAK2^V617F^ Mutated Cells from Primary Myelofibrosis Patients

**DOI:** 10.3390/ijms21082926

**Published:** 2020-04-22

**Authors:** Michelino Di Rosa, Cesarina Giallongo, Alessandra Romano, Daniele Tibullo, Giovanni Li Volti, Giuseppe Musumeci, Ignazio Barbagallo, Rosa Imbesi, Paola Castrogiovanni, Giuseppe A. Palumbo

**Affiliations:** 1 Department of Biomedical and Biotechnological Sciences, Human Anatomy and Histology Section, School of Medicine, University of Catania, 95125 Catania, Italy; roimbesi@unict.it (R.I.); pacastro@unict.it (P.C.); 2 Department of Medical, Surgical Sciences and Advanced Technologies “G.F. Ingrassia”, University of Catania, 95125 Catania, Italy; cesarinagiallongo@yahoo.it (C.G.); giuseppealberto.palumbo@gmail.com (G.A.P.); 3 Division of Hematology, A.O.U. Policlinic-OVE, University of Catania, 95122 Catania, Italy; sandrina.romano@gmail.com; 4 Department of Biomedical and Biotechnological Sciences, Medical Biochemistry Section, University of Catania, 95125 Catania, Italy; d.tibullo@unict.it (D.T.); livolti@unict.it (G.L.V.); 5 Research Center on Motor Activities (CRAM), University of Catania, 95125 Catania, Italy; giumusu@gmail.com; 6 Department of Drug Science, Biochemistry Section, University of Catania, 95125 Catania, Italy; ignazio.barbagallo@unict.it

**Keywords:** immunoproteasome, JAK2^V617F^, primary myelofibrosis, bioinformatics, CD34^+^ cells, HLA-class I, innate immunity

## Abstract

Primary myelofibrosis (PMF) is a rare myeloproliferative neoplasm characterized by stem-cell-derived clonal over-proliferation of mature myeloid lineages, bone marrow fibrosis, osteosclerosis, defective erythropoiesis, and pro-inflammatory cytokine over-expression. The aim of the present study was to highlight possible differences in the transcriptome among CD34^+^ cells from peripheral blood (PB) of PMF patients. Therefore, we merged two microarray datasets of healthy control subjects and PMF (34 JAK2^V617F^ MUTATED and 28 JAK2 wild-type). The GO analysis of upregulated genes revealed enrichment for JAK2/STAT1 pathway gene set in PB CD34^+^ cells of PMF patients with and without the *JAK2^V617F^ mutation* comparing to the healthy control subjects, and in particular a significant upregulation of immunoproteasome (IP)-belonging genes as *PSMB8*, *PSMB9,* and *PSMB10.* A more detailed investigation of the IFN-gamma (IFNG) pathway also revealed that *IFNG, IRF1,* and *IFNGR2* were significantly upregulated in PB CD34^+^ cells of PMF patients carrying the mutation for JAK2^V617F^ compared to JAK2 wild-type PMF patients. Finally, we showed an upregulation of HLA-class I genes in PB CD34^+^ cells from PMF JAK2^V617F^ mutated patients compared to JAK2 wild-type and healthy controls. In conclusion, our results demonstrate that IPs and IFNG pathways could be involved in PMF disease and in particular in patients carrying the *JAK2^V617F^ mutation*.

## 1. Introduction

Primary Myelofibrosis (PMF) is a Philadelphia-negative myeloproliferative neoplasm (MPNs), characterized by stem cell-derived clonal proliferation of one or more myeloid lineage cells [1]. It is associated with bone marrow fibrosis, osteosclerosis, angiogenesis, extramedullary hematopoiesis, and adnormal cytokine levels [2]. Most patients with PMF carry one of three mutually exclusive somatic driver mutations JAK2^V617F^ (about 60%) [3], Calreticulin (CALR) (about 20%) [4] and MPL (about 5%) [5,6]. These genetic markers have been recently included in the major diagnostic criteria for PMF, and the presence of JAK2^V617F^ mutant confers an inferior outcome than the CALR mutant. More recently, the molecular landscape of PMF has become increasingly well characterized, leading to the development of genetically-based prognostic scoring systems such as MIPPS70, MIPSS70^+^ version 2.0, and GIPPS [7,8,9]. Before the discovery of JAK2 mutation, one of the most important prognostic factors of evolution toward blast transformation in PMF was the absolute number of circulating CD34^+^ cells in peripheral blood [10]. In the post-genomic era, CD34^+^ cells in peripheral blood >10/μL can still distinguish PMF from other MPNs with high sensitivity and specificity [6]. PMF patients have few therapeutics options because there is limited information on its biology. Ruxolitinib (RUX) is a first-in-class oral JAK1/JAK2 inhibitor approved for the treatment of patients with myelofibrosis based on the results of two randomized clinical trials (COMFORT-I and COMFORT-II) [11]. Clinical benefits of RUX are partially derived from the reduction of inflammatory cytokines, with an early relief of clinical symptoms and reduction of spleen size after 4 weeks post-treatment [12,13]. Only in a few cases the drug reverts bone marrow fibrosis or reduces the allele burden [14], suggesting that other intracellular signaling in the neoplastic clone or in the host-tumor interaction can affect the clinical course of PMF.

Several papers have shown evidence of a dysregulation of the immune system in the MPNs. PMF is considered as an inflammatory disease where the higher cytokine secretion creates a pro-inflammatory milieu influencing the immune system [15]. It has been demonstrated that several immune defects are principally associated with the presence/absence of the *JAK2^V617F^ mutation* [16,17]. Overall, these anomalies could contribute to the development of an immune deficiency state with the potential to promote immune evasion, cancer progression and increased susceptibility to infections [18]. Furthermore, a better understanding of immune biology in the context of PMF would be important for the design of new therapies for PMF.

In eukaryotic cells, the proteasomes (c-20S) are ubiquitously-expressed cellular proteases involved in the degradation of intracellular oxidized proteins following an oxidative insult, through an ATP-independent mechanism [19]. Being ubiquitously expressed, these proteins represent a potential pharmacological target even though with several limitations [20]. To this regard, Bortezomib, a potent and clinically relevant proteasome inhibitor, is intermittently used for multiple myeloma treatment (MM) [21,22] and other inflammatory disease [23,24,25], in order to limit toxic effects [26]. In cells of hematopoietic origins, the classical proteasome is replaced by a different proteasome with an immunological role called immunoproteasome (IPs) [27]. The origin of this term arises from the fact that it was discovered during studies of antigen presentation on the cell surface for T-cell recognition to stimulate the immune response in collaboration with major histocompatibility class I (MHC class I) molecules. Both innate immunity (lymphocytes) and acquired immunity (monocytes, dendritic cells, and macrophages) [28] cells during inflammatory processes express the 20s immunoproteasome subunits (i-20) [29]. Additionally, stimulation with type I Interferon [30], Tumor Necrosis Factor alpha (TNFα) [31], or IFNG [32], cytokines that are essential for both innate and adaptive immune response to viral and bacterial infections, stimulates new i-20S. Considerable interest has been focused on developing immunoproteasome-specific inhibitors (IPSIs) for applications in autoimmune disorders such as systemic lupus erythematosus [33], inflammatory bowel disease [34], and rheumatoid arthritis [35]. The i-20S proteasome is generally expressed in the spleen, thymus, bone marrow, and lymph nodes, all of which are associated with lymphocyte maturation [36]. Furthermore, the proteasome inhibition also represents an attractive potential anticancer therapy. Since Bortezomib was able to inhibit the NF-kappaB pathway in MM [21], it was believed that it could also be effective for PMF patients. However, the first clinical studies on PMF patients did not show encouraging results [37], although the pre-clinical results on the mouse model seemed very promising, having determined a decrease in the transformation of growth factor-β1 and osteoprotegerin levels, a reduction in osteosclerosis, and as a direct consequence an increase in survival [38]. Lack of clinical efficacy of Bortezomib in myelofibrosis may be linked to the need for blocking oncogenic driver mutations including Janus Kinase 2 and Calreticulin.

With the aim of identifying new possible molecular targets, we used the datasets available in GEODataset [39] in order to describe the main differences in the transcriptome of CD34^+^ hematopoietic progenitor cells circulating in peripheral blood (PB) of healthy individuals, and in wild-type or JAK2^V617F^ mutated PMF patients, trying to draw a starting line for future investigations.

## 2. Results

### 2.1. Identification of Potential Genes Modulated in JAK^2V617F^ Mutated Compared to JAK2 Wild-Type PMF Patients

**Figure 1:**
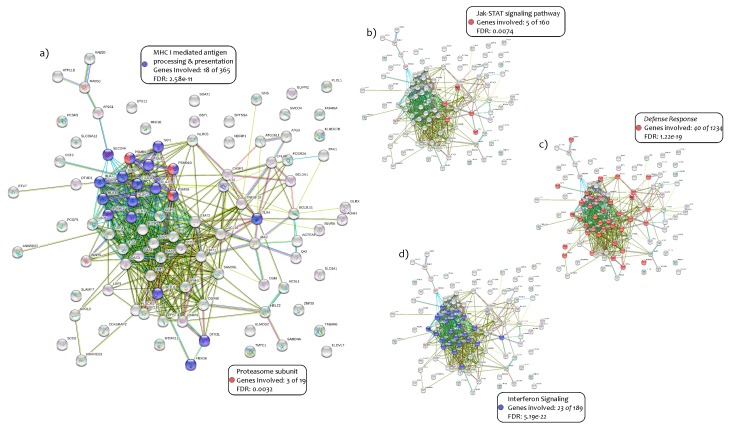
GO analysis in 100 genes upregulated in JAK2^V617F^ mutated patients.The GO analysis performed with the online tool GeneMANIA and GHATER showed the following results: 18 genes out of 365 belonging to the pathway of MHC class I-mediated antigen processing and presentation (*p* = 2.50 × 10^−11^) (**a**); 3 genes out of 19 belonging to the Immunoproteasome (IPs) (*p* = 0.0032) (a); the involvement of JAK-STAT signal pathways (*p* = 0.0074) (**b**); the Immuno-Defense-Response (40 out of 1234 genes) (*p* = 1.22 × 10^−19^) (**c**); 23 genes out of 189 belonging to the IFNG pathways (*p* = 5.19 × 10^−22^) (**d**).

From microarray datasets, we selected 34 PMF patients carrying the *JAK2^V617F^ mutation* and 28 JAK2 wild-type patients. We compared the two groups of study and obtained 1278 upregulated and 2070 downregulated genes in JAK2^V617F^ mutated patients compared to the JAK2 wild-type (Supplementary Table S1). A Gene Ontology (GO) analysis performed on the first 100 most significant modulated genes (*p* < 0.0001) showed impressive results (Figure 1) (Supplementary Table S1). Then we identified 18 genes out of 365 (4.9%) belonging to the pathway of MHC class I mediated antigen processing and presentation (*p* = 2.58 × 10^−11^) and three genes out of 19 (15.7%) belonging to the Immunoproteasome (IPs) (*PSMB8, PSMB9 and PSMB10*) (*p* = 0.0032) (Figure 1a) (Supplementary Table S1).

Furthermore, as expected, the involvement of JAK-STAT signaling pathways in JAK2^V617F^ mutated patients with the transcription of *JAK2, STAT1, STAT2* and *OSM* genes was confirmed (*p* = 0.0074) (Figure 1b) (Supplementary Table S1). A large number of genes belonging to the Immuno-Defense-Response were highlighted (40 out of 1234 genes) (3.2%) (*p* = 1.22 × 10^−19^) (Figure 1c) (Supplementary Table S1). Moreover, the IFNG signaling pathways were significantly involved through the expression of 23 genes out of 189 available (*p* = 5.19 × 10^−22^, 12.1%) (Figure 1d) (Supplementary Table S1). In our analysis, we also observed several antiviral response activated pathways, such as the double-strand *RNA* virus *(OAS1, OAS2 and OAS3)* (three out of nine genes; *p* = 0.00066, 33.3%) [40,41,42,43,44], the Herpes simplex (14 out of 181 genes; *p* = 1.10 × 10^−10^, 7.7%), and Influenza A virus (10 out 168 genes; *p* = 5.29 × 10^−7^, 5.9%) (Supplementary Table S1). These specific activated pathways could be due to the IFNG signaling.

### 2.2. Immunoproteasome (IPs) Genes Expression in PMF Patients

**Figure 2:**
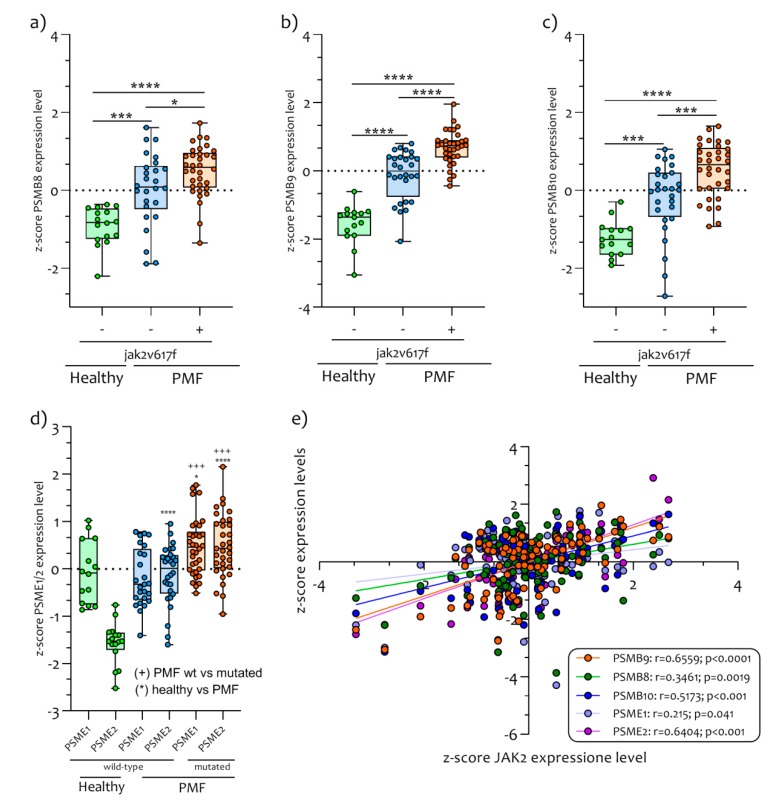
IPs genes expression in PB CD34^+^ Cells of JAK2^V617F^ mutated/wild-type PMF patients and healthy control subjects. The genes belonging to the IPs family are *PSMB8* (**a**), *PSMB9* (**b**), *PSMB10* (**c**) and *PSME1/2* (**d**). The patients affected by primary myelofibrosis express significant upregulated levels of IPs compared to healthy controls. In addition, the PMF patients mutated for JAK2^V617F^ express significant upregulated levels of IPs genes compared to wild-type patients. The *JAK2* gene expression levels in PB CD34^+^ cells of PMF patients were significantly correlated with IPs genes expression. Data are expressed as z-score intensity expression levels and presented as vertical scatter dot plots. *p* values < 0.05 were considered to be statistically significant (* *p* < 0.05; *** *p* < 0.0005; **** *p* < 0.00005).

Our analysis showed that *PSMB9* is the most significantly modulated gene in PB CD34^+^ cells of PMF patients, both with and without *JAK2^V617F^* mutation, compared with healthy subjects (*p* < 0.00001) (Figure 2) (Supplementary Table S1).

**Figure 3:**
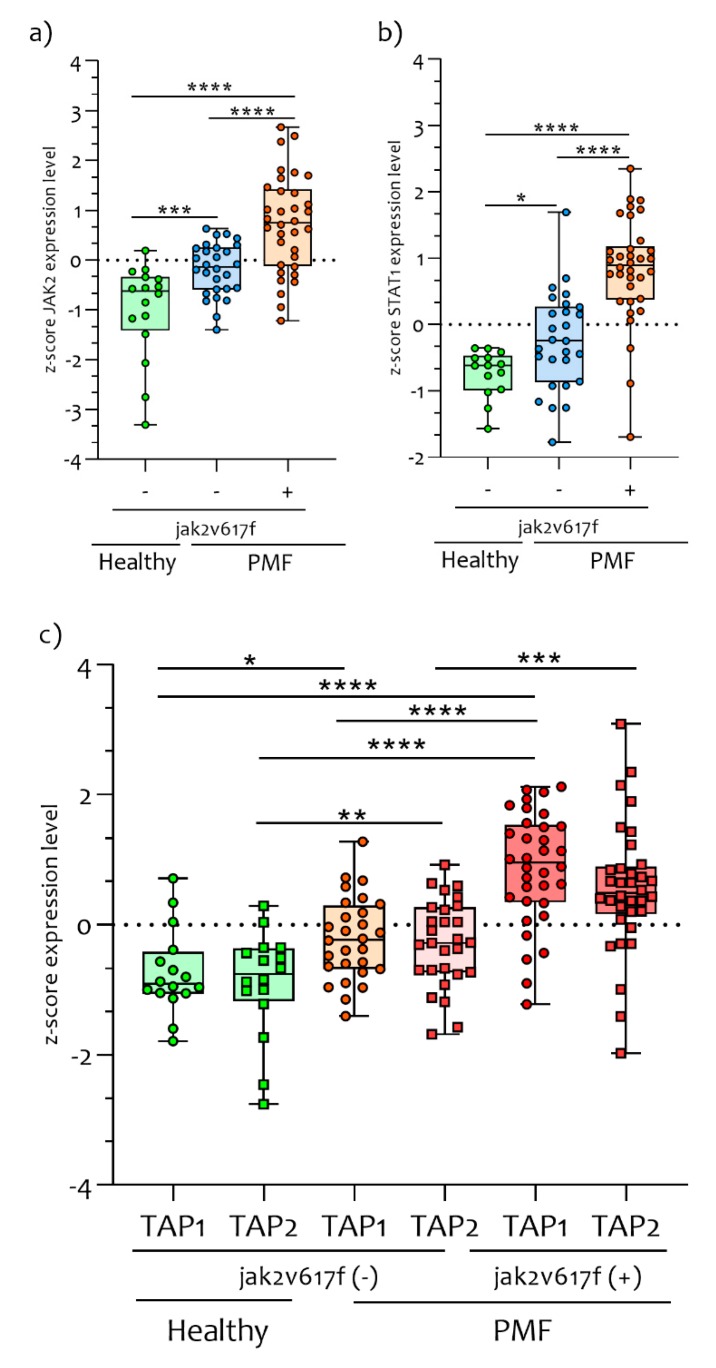
*JAK2/STAT1* and *TAPs* gene expression levels in PMF patients and healthy control subjects. The *JAK2/STAT1* expression levels are closely linked to the PMF condition. During our analysis, we showed that *JAK2/STAT1* gene expression levels were significantly upregulated in PB CD34^+^ cells of PMF patients compared to healthy controls subjects (**a** and **b**). Furthermore, the PMF patients who had the *JAK2^V617F^* mutation had significantly upregulation of *JAK2*/*STAT1* compared to wild-type. Transporters associated with Antigen Processing 1 and 2 are proteins that in humans are encoded by the *TAP1* and *TAP2* genes. These genes are involved in the degradation of peptides in order to assemble at the class I molecules. In CD34^+^ cells of PMF patients, we showed that *TAP1* and *TAP2* expression levels were significantly upregulated compared to healthy controls subjects (**c**). Furthermore, PMF JAK2^V617F^ mutated patients presented significantly upregulated levels of *TAP1* and *TAP2* compared to PMF JAK2^V617F^ wild-type. Data are expressed as z-score intensity expression levels and presented as vertical scatter dot plots. *p* values < 0.05 were considered to be statistically significant (* *p* < 0.05; ** *p* < 0.005; *** *p* < 0.0005; **** *p* < 0.00005).

This gene, together with another two catalytic subunits (PSMB8 and PSMB10), constitute most of the IPs that are constitutively expressed in hematopoietic cells and induced by pro-inflammatory cytokine such as IFNG. Furthermore, the expression of *PSMB8* (*p* < 0.00001), *PSMB9* (*p* < 0.00001), *PSMB10* (*p* < 0.00001) and *PA28*α and β (*PSME1* and *PSME2* respectively) (Figure 2) were significantly upregulated in JAK2^V617F^ mutated PMF patients compared to healthy JAK2^V617F^ wild-type PMF and to controls subjects (*PSMB8*, *p* < 0.00001; *PSMB9*, *p* < 0.00001; *PSMB10*, *p* < 0.0001; *PSME1*, *p* = 0.0001; *PSME2*, *p* < 0.0001) (Figure 2a–d). We also showed that all IPs genes were significantly correlated to the *JAK2* gene expression levels (*PSMB8* r = 0.3461, *p* = 0.0019; *PSMB9* r = 0.6559, *p* < 0.0001; *PSMB10* r = 0.5173, *p* < 0.001; *PSME1* r = 0.215, *p* = 0.041; *PSME2* r = 0.6404, *p* < 0.0001) (Figure 2e). Furthermore, we observed that *JAK2* and *STAT1* genes expression levels were significantly modulated in JAK2^V617F^ mutated PMF patients compared to PMF JAK2^V617F^ wild-type (*JAK2*, *p* < 0.00001 and *STAT1*, *p* < 0.00001) and to healthy controls (*JAK2*, *p* < 0.00001 and *STAT1*, *p* < 0.00001) (Figure 3a,b).

Significant differences in *JAK2* (*p* = 0.0007) and *STAT1* (*p* = 0.024) expression levels were observed by comparing healthy controls to PMF patients who are wild-type for the *JAK2^V617F^ mutation*. These results seem to indicate an activation of the JAK2 pathway in both mutated and wild-type PB CD34^+^ cells of PMF patients (Figure 3a,b).

Deeping our investigation on IPs pathways, we observed a significant upregulation in the expression levels of *TAP1* (*p* < 0.01) and *TAP2* (*p* < 0.001) in PB CD34^+^ cells of PMF patients compared to healthy controls subjects (Figure 3c). Furthermore, the *TAP1* (*p* < 0.00001) and *TAP2* (*p* < 0.0001) expression levels were significantly upregulated in JAK2^V617F^ mutated compared to JAK2 wild-type patients (Figure 3c). Significant differences in *TAP1* (*p* < 0.00001) and *TAP2* (*p* = 0.0003) expression levels were observed by comparing PMF patients with and without *JAK2^V617F^* mutation (Figure 3c).

Furthermore, we verified the trend of the constituent isoforms of the proteasome (PSMB5, PSMB6, and PSMB7). None of the three components showed significant changes between JAK2^V617F^ mutated PMF patients and wild-type patients (Figure S1a). The gene expression levels of the *PSMB5* and *PSMB6* subunits were significantly increased in PMF patients, regardless of the presence of the mutation. With regards to the gene expression levels of the *PSMB7* subunit, they were downregulated in PMF patients, effectively preventing the formation of the proteasome complex in PMF patients. To confirm this, the expression levels of the *PSMB7* subunit were inversely related to the expression of *JAK2* (Figure S1b).

### 2.3. IFNG Pathways Activation in PB CD34^+^ Cells of PMF Patients

**Figure 4:**
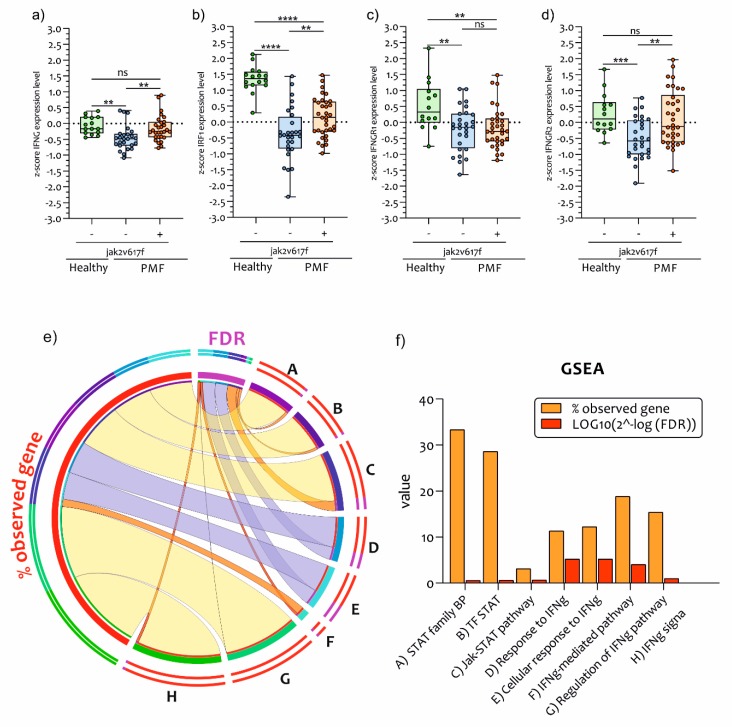
*IFNG* genes pathways expression levels in PB CD34^+^ Cells of PMF patients. *JAK2* is necessary and sufficient for IFNG-induced transcription of genes involved in immuno-response against antigens self/non-self. In our analysis, we showed that *IFNG* gene expression levels were significantly downregulated in PB CD34^+^ cells of PMF patients wild-type for *JAK2^V617F^ mutation* compared to healthy controls subjects (**a**), but not compared to JAK2^V617F^ mutated patients. Similar trends were observed for *IRF1* (**b**) and *IFNGR2* (**d**). The *IFNGR1* modulation was an exception (**c**). GSEA of IFNG and STAT pathways. The data visualization was obtained by tool CIRCOS. The ribbons are expressed by weighted percentage (Q1 = red; Q2 = orange; Q3 = yellow; Q4 = purple). For Q1 and Q2 we used a transparency of 4 and no stroke (**e**). GSEA of *IFNG* and *STAT1* pathways are expressed in a bar chart (**f**). Data are expressed as z-score intensity expression levels and presented as vertical scatter dot plots. *p* values < 0.05 were considered to be statistically significant (** *p* < 0.005; *** *p* < 0.0005; **** *p* < 0.00005).

Our GO analysis highlighted the activation of the IFNG pathway. We have further investigated the gene expression levels of *IFNG*, *IRF1*, and *IFNGR1/2* gene expression. We have showed that there was a significant downregulation in *IFNG* (*p* < 0.001) expression levels in JAK2 wild-type PMF PB CD34^+^ cells compared to healthy controls subjects. Patients presenting the *JAK2^V617F^ mutation* had significant upregulation of *IFNG* (*p* < 0.001) expression levels compared to PMF JAK2^V617F^ wild-type patients (Figure 4a).

The same trend was observed for both the expression levels of *IRF1* (*p* = 0.0073) and *IFNGR2* (*p* = 0.015) (Figure 4b,d) (Supplementary Table S1). By contrast, *IFNGR1* expression was significantly downregulated in JAK2 wild-type (*p* = 0.0033) and mutated (*p* = 0.0027) CD34^+^ PMF patient PB cells compared to healthy subjects, while there was no significant difference between JAK2 wild-type and mutated patients (*p* = 0.8148) (Figure 4c). This data was confirmed by GSEA for IFNG and STAT1/2 pathways (Figure 4e,f). About 33.3% of the genes involved in STAT1-activated pathways were upregulated in PMF JAK2 mutated patients. Furthermore, the IFNG pathways were significantly modulated (FDR = 5.21 × 10^−18^) in mutated PMF patients compared to wild-type PMF.

**Figure 5:**
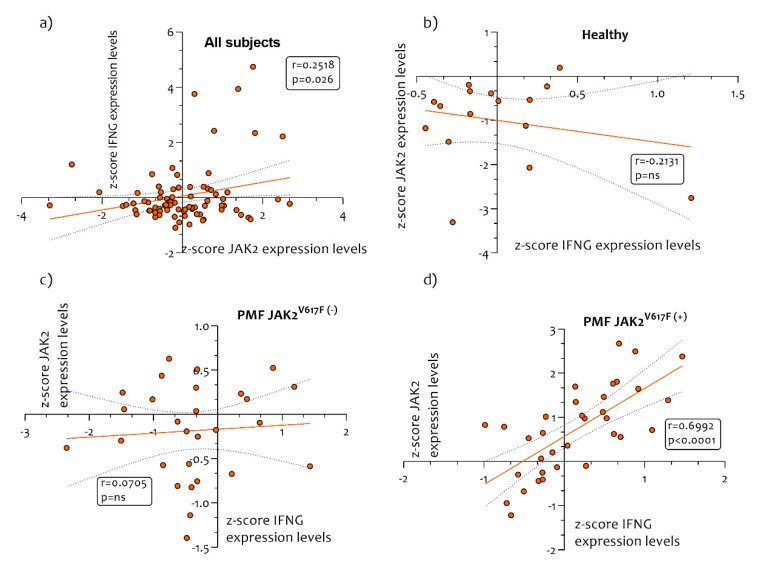
Correlation analysis between *IFNG/JAK2* expression levels. A Pearson correlation analysis was performed in order to verify the potential correlation between *JAK2* and *IFNG* in healthy controls subjects and in PMF patients. We showed a positive correlation (r = 0.2518, *p* = 0.026) in all the subjects recruited in the study between *IFNG/JAK2* expression levels (**a**). No correlation was observed in healthy controls subjects (**b**) and in PMF patient’s wild-type for *JAK2^V617F^ mutation* (B/C). A significantly positive correlation was observed in PMF patients mutated for JAK2^V617F^ (r = 0.6992, *p* < 0.0001) (**c**). *p* values < 0.05 were considered to be statistically significant.

In order to verify whether *IFNG* gene expression was dependent on JAK2 expression, we performed a Pearson correlation analysis. We showed a positive correlation (r = 0.2518, *p* = 0.026) in all subjects recruited in the study between *IFNG* versus *JAK2* expression levels (Figure 5a).

Furthermore, no correlation was observed in healthy control subjects and in PMF patient who were wild-type for *JAK2^V617F^ mutation* (Figure 5b,c). However, we observed a positive correlation between the expression levels of *JAK2* and *IFNG* in PMF patients mutated for JAK2^V617F^ (r = 0.6992, *p* < 0.0001) (Figure 5c).

### 2.4. Antigen Exposition Pathways in PB CD34^+^ Cells of PMF Patients

**Figure 6:**
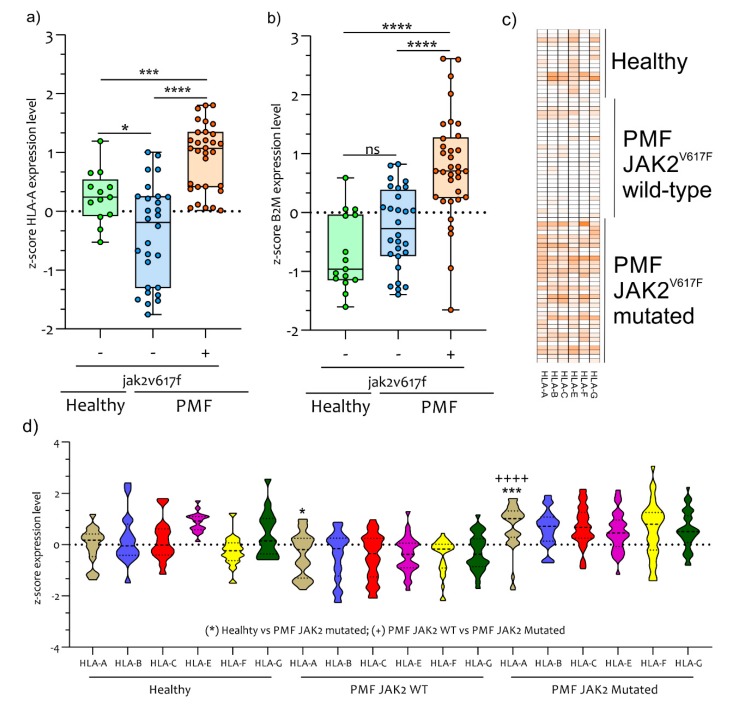
*HLA-A* and *B2M* genes expression are modulated in PB CD34^+^ Cells of PMF patients. *HLA-A* expression levels clearly differed between PMF patients with *JAK2^V617F^ mutation* compared to wild-type patients and healthy control subjects. A significant upregulation was observed in *HLA-A* expression levels (**a**) and in B2M (**b**) in PMF patients mutated for JAK2^V617F^ compared to wild-type patients and healthy controls subjects. Heatmap of *HLAs class I gene* expressed in PB CD34^+^ Cells of healthy, PMF patients JAK2 mutated and wild-type (**c**). Z-score expression levels of *HLAs class I gene* in in PB CD34^+^ Cells of healthy, PMF patients JAK2 mutated and wild-type (**d**). Data are expressed as z-score intensity expression levels and presented as vertical scatter dot plots and violin plot. *p* values < 0.05 were considered to be statistically significant (* *p* < 0.05; *** *p* < 0.0005; **** *p* < 0.00005).

Following IFNG, JAK2, and IPs pathway activation we further investigated the possible involvement of the MHC class I (HLA class I)-mediated recognition system consistently with the GO analysis (Figure 1). In order to test this hypothesis, we analyzed the *HLA* family genes expression in PMF patients compared to healthy controls subjects. Our analysis showed a significant upregulation in the expression levels of the *HLA-A, HLA-B, HLA-C, HLA-E, HLA-F* and *HLA-G* genes in PB CD34^+^ cells from PMF JAK2^V617F^ mutated patients compared to JAK2 wild-type patients and healthy control subjects (Figure 6a–d). In particular, we showed a significant upregulation in the expression of *HLA-A* (the most modulated gene among the *HLAs* genes) (Figure 6a,c,d) and *B2M* (Figure 6b) in PB CD34^+^ cells from JAK2^V617F^ mutated PMF patients compared to *JAK2* wild-type patients (*HLA-A*, *p* < 0.00001 and *B2M p* < 0.00001) and healthy controls subjects (*HLA-A*, *p* < 0.0001 and *B2M, p* < 0.00001).

In addition, JAK2^V617F^ wild-type PMF patients presented a significant downregulation of *HLA-A* expression levels compared to healthy controls subjects (*p* < 0.01) (d). No significant modulation was observed in *B2M* expression levels between healthy controls subjects and JAK2^V617F^ wild-type PMF patients (*p* = 0.056) (Figure 6b). Finally, compared to healthy controls, CD34^+^ cells from PMF patients showed significative up-regulation of *ARG1,* which correlated with the *JAK2* expression levels in JAK2^V617F^ mutated patients (r = 0.4181 and *p* = 0.0139) (Figure S2a–c).

### 2.5. Diagnostic Accuracy of mRNA PSMB8, PSMB9, and PSMB10 for PMF Patients

**Figure 7:**
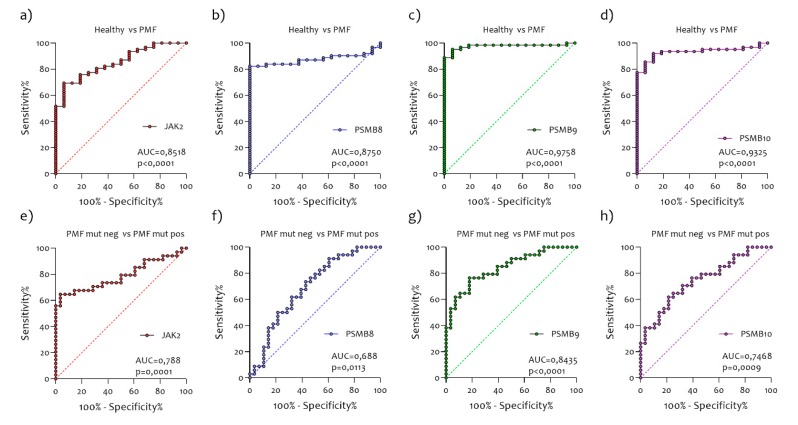
Diagnostic accuracy of *JAK2* (**a**,**e**), *PSMB8* (**b**,**f**), *PSMB9* (**c**, **g**), and *PSMB10* (**d**, **h**) mRNA for PMF patients. For single predictors, *PSMB9* (c) had the highest accuracy, followed by *PSMB10* (d) and *PSMB8* (b). For PMF wild-type versus PMF mutated, *PSMB9* (g) is a significant individual predictor.

**Figure 8:**
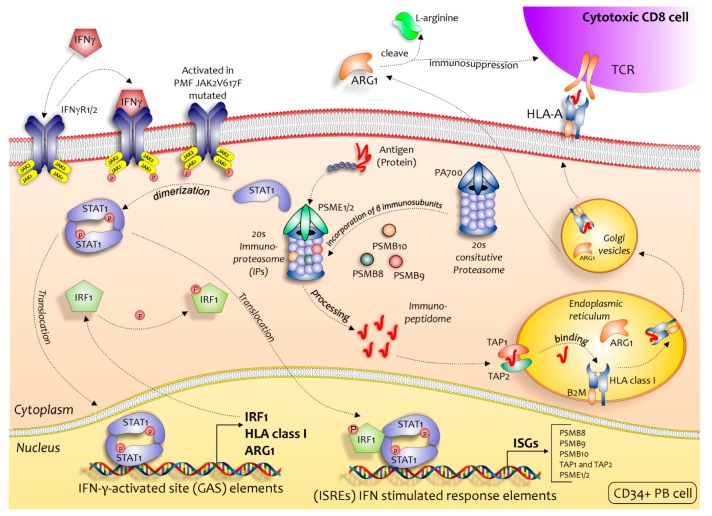
Regulatory role of the IPs in immune surveillance in PB CD34^+^ Cells of PMF JAK2^V617F^ mutated patients. The activation of JAK2 pathway, in a classical manner through the binding of IFNG to its receptor (IFNGR1/2) or through the *JAK2^V617F^ mutation*, induces the expression of the immunoproteasome-specific subunits β1i (PSMB9), β2i (PSMB10), β5i (PSMB8), PA28 (PSME1), and PA28 (PSME2) (via STAT1 and IRF1) that result in the preferred assembly of the immunoproteasome over the regular proteasome. The resulting immuno-peptidome more effectively binds to MHC class I molecules (induced during the activation of JAK2), such that after processing in the endoplasmic reticulum (ER) (TAP1 and TAP2) and Golgi apparatus the individual peptides presented on the cell surface can be recognized by T-cell receptors on CD8^+^ cells, initiating an immune response (increased ARG1 expression is known to result in arginine deficiency, which leads to immunosuppression by impairing lymphocyte proliferation and activation).

We tested accuracies for PMF wild-type patients versus PMF mutated and versus healthy using logistic regression models (Figure 7). *PSMB8* (AUC = 0.8750), *PSMB9* (AUC = 9758), and *PSMB10* (AUC = 0.9325) were all significant predictors of PMF, all with a higher AUC than *JAK2* (AUC = 0.8518) (Figure 7a–d). For single predictors, *PSMB9* had the highest accuracy, followed by *PSMB10* and *PSMB8*. For PMF wild-type versus PMF mutated, PSMB9 is a significant individual predictor (AUC = 0.8435) (Figure 8g), while *JAK2* (AUC = 0.788) (Figure 8e) was the following predictor (*PSMB8* AUC = 0.688, *PSMB10* AUC = 0.7468) (Figure 7f,h).

## 3. Discussion

In this manuscript, we hypothesized that there are significant differences in transcriptomes among PB CD34^+^ cells of PMF patients with and without *JAK2^V617F^ mutation*. To address our hypothesis, we merged two microarray datasets available on a GEO Dataset, for a total of 31 healthy control subjects and 62 PMF patients (34 with and 28 without *JAK2^V617F^ mutation*). Our transcriptome analysis of circulating CD34^+^ cells wild-type and JAK2^V617F^ mutated PMF patients disclosed a dysregulation in the antigen presentation signaling involving immunoproteasome and upregulation of HLA class I genes. As expected, in JAK2^V617F^ circulating CD34^+^ cells there was an involvement in the JAK2/STAT1 and IFNG gene pathways.

During inflammatory processes, in response to stimulation with type I interferon [45], TNFα [31], or IFNG [46], cells of hematopoietic origins can replace the c-20S with the so-called immunoproteasome (i20S) [27], as discovered during studies of antigen presentation on the cell surface for T-cell recognition to stimulate the immune response in collaboration with major histocompatibility class I (MHC class I) molecules. With the expression of the i-20s subunit, the standard catalytic subunits 1, 2 and 5 of c-20s are substituted with the subunits 1_i_ (LMP2 or PSMB9), 2_i_ (MECL-1 or PSMB10), and 5_i_ (LMP7 or PSMB8) respectively [47]. Once the IP was activated, this promoted helper T (Th) cell differentiation (including pro-inflammatory Th1 and Th17 cells) and effector T cell expansion (cytotoxic CD8 cells), while repressing regulatory T (Treg) cell induction, admittedly through yet unidentified pathways [48]. It has been observed that IPs inhibition suppresses the expression of the pro-inflammatory IFNG, TNF-α, GM-CSF, and IL-6 cytokines in activated T cells [49]. All these cytokines are highly expressed in PB of PMF patients and might play a key role in the progression of the disease [50,51,52]. These evidences are in accordance with our results.

Recently, numerous transcriptome analyses have been performed to study the main Philadelphia-negative myeloproliferative neoplasms (MPNs). These analyses have shown promising new target genes for the various pathologies examined. The impairment of the immunologic framework has emerged almost always, showing an alteration of the pro-inflammatory, pro-differentiation and anti-apoptotic transcription lines. As an example, in idiopathic myelofibrosis (IM) [53], and in PMF [54,55,56] it emerged from the analyses that the WT1 gene is highly modulated. A T-cell receptor (TCR) that specifically reacts with WT1 peptide in the context of HLA-A * 24:02 has been identified [57]. The receptor recognition mechanism passes through the activation of the IP. As shown recently by Stetka et al. [58], numerous genes belonging to immuno-activation have been identified to be highly modulated in PV. In this manuscript, the authors treated the JAK2 wild-type and JAK2^V617F^ CD34+ progenitors with medium without or with inflammatory cytokines (IFN-gamma, TNF-α, and TGF-β). They showed that among the top 20 differentially overexpressed genes shared by both types of progenitors (JAK2 wild-type and V617F mutant) treated with inflammatory factors are some members from our gene set of differentially expressed genes, including *IRF1, STAT1, B2M,* and *TAP1*. These data suggest that the expression signature characterizing the JAK2^V617F^ mutated PMF patients may result not only from intrinsic V617F-driven expression program, but also from extrinsic inflammatory cytokine-driven activation, which could be different in JAK2^V617F^ mutated and JAK2 wild-type PMF.

Immune dysfunction in PMF is an intriguing emerging field [59]. Differently from other MPNs [60,61], T-cells count is preserved, but there is an altered regulatory T cell frequency, expansion of myeloid-derived suppressor cells, and CD4/natural killer cell dysfunction [62], while data on CD8^+^ are lacking. Our analysis would suggest that, based on interaction with hematopoietic progenitors, CD8 T cells are more active in patients with PMF. This is in line with the clinical observation that hematopoietic progenitors could respond to chronic inflammation in the context of a systemic autoimmune disease favoring fibroblast activation leading to bone marrow fibrosis and progressive cytopenias, in both primary and secondary myelofibrosis [63]. Moreover, we found that HLA class I genes expression levels were closely related to PMF disease and *JAK2^V617F^ mutation*, indicating a potential relationship with CD8^+^ T-cells [64]. The downregulation of class I and II HLA genes is used by tumor cells to escape antitumor T-cell-mediated immune responses. Although the expression levels of *HLA* genes are high in MPNs, there is no evidence in PMF patients [65,66]. The upregulation of HLA class I genes is important for tumor immune surveillance by IFNG treatment in PMF. This mechanism might enhance the cytotoxic potential of immune cells against PMFs. Unfortunately, as mentioned above there is no available data that indicate CD34^+^ PMF cells as a potential target for the cytotoxic action of CD8^+^ T cells.

From our analysis, it seems that the *JAK2^V617F^ mutation* increases, on one hand, the capacity of immunological response with the activation of IPs pathways, but at the same time the immune response against CD34^+^ PMF cells seems to be ineffective. We could speculate that elevated endoplasmic reticulum stress induces the release of damage-associated molecular patterns to the tumor microenvironment, which activates IFN-gamma signaling in PMF cells. The elevated IFN-gamma signaling induces higher activity in the IPs, which might improve antigen presentation and result in the recruitment of TILs to the bone marrow, the release of pro-inflammatory cytokine and as a consequence, the increase of fibrosis [67]. In this regard, the activation of IPs determines the ability of the PMF cells to be potentially recognized by cytotoxic CD8^+^ cells (Figure 8).

Knowing this, blocking the IPs action could have direct repercussions on the pro-inflammatory cytokines expression levels in PMF and subsequently on its clinical course, making IPs an extremely interesting candidate in the search for anticancer drugs.

IP modulation is extremely varied in the different types of tumors, since IPs can be upregulated in some scenarios (e.g., prostate cancer and lung cancer) [68,69] and down-regulated in others (e.g., colon [70], kidney, skin, neck, head, and esophagus) [20]. The selective i-20S inhibitor carfilzomib has shown clinical activity in vitro in primary CD34^+^ PMF cells and it is safe in combination with ruxolitinib in patients affected by several hematological malignancies (clinical trial.gov, NCT03773107), suggesting that targeting IPs is worth being investigated in PMF. We must bear in mind that, abnormal MHC class I expression and the loss of antigen processing are features of malignant cells [71]. T cell‒mediated immune tumor suppression is a complex process with numerous requirements, among which is antigen processing by the IPs and presentation through MHC class I surface molecules expressed on tumor cells. The IPs genes are regulated by both cell-intrinsic and cell-extrinsic factors in different types of cancer. In PMF patients, regardless of the *JAK2^V617F^ mutation*, it would appear that the entire IPs activation pathways are transcribed. In fact, from our analysis, it was shown that ARG1 was also upregulated in PMF patients compared to healthy control subjects.

An interesting hypothesis to be demonstrated of the activation of the IP in the PMF could be the presence of a previous viral infection underlying the activation of IPs [72]. In this case, the MHCs class I would carry viral antigens ready to be recognized, but due to the immune escape activated by the tumor cells, with the production of ARG1, LAG3, and CTLA4 (Supplementary Table S1), they do not recognize it.

## 4. Materials and Methods

### 4.1. Data Selection

The NCBI Gene Expression Omnibus (GEO) database [73] was used to select transcriptome datasets to analyze genes expression in primary myelofibrosis (PMF) patients. Mesh terms “myelofibrosis”, “JAK2^V617F^”, “JAK2”, “CD34^+^” and “Human” were used to identify potential datasets of interest. We sorted the obtained datasets by the number of samples (High to Low), age, gender, and for clinical data made available by the authors. Because of the few PMF studies, only two datasets (GSE53482, GSE41812,) were selected. A total of 78 samples (16 healthy control and 62 PMF patients) were analyzed. Data sample collection are available in Table 1. Supplementary information of the sample recruited are available in Series Matrix File (s).

The GSE53482 (platform GPL13667), was composed of Peripheral Blood (PB) CD34^+^ Cells from 16 healthy donors and 42 PMF patients (23 PMF patients carrying the mutation JAK2^V617F^ and 19 JAK2 wild-type samples) [74]. We selected data from GSE41812 (platform GPL13667) relative to PB CD34^+^ cells of 20 PMF patients (11 carrying the mutation JAK2^V617F^, and 9 were wild-type) [75].

### 4.2. Data Processing and Experimental Design

In order to process and identify Significantly Different Expressed Genes (SDEG) in all selected datasets, we used the MultiExperiment Viewer (MeV) software (The Institute for Genomic Research (TIGR), J. Craig Venter Institute, La Jolla, USA). In cases where multiple genes probes insisted on the same GeneID, we used those with the highest variance. The significance threshold level for all data sets was *p* < 0.05. The genes with *p* < 0.05 were identified as SDEG and selected for further analysis. For all datasets we performed a statistical analysis with GEO2R, applying a Benjamini & Hochberg (False discovery rate) to adjust *p* values for multiple comparisons [42,76,77,78].

From all datasets, we performed a comparison analysis of significantly expressed genes in PB CD34^+^ Cells from PMF patients carrying the mutation JAK2^V617F^ compared to JAK2 wild-type PMF patients. We obtained 1278 upregulated and 2070 downregulated genes (Supplementary Table S1) in JAK2^V617F^ mutated patients compared to JAK2 wild-type. The genes ontology analysis was performed using the web utility GeneMANIA [79] and GHATER (Gene Annotation Tool to Help Explain Relationships) [80] (Supplementary Table S1). 

### 4.3. Statistical Analysis

For statistical analysis, Prism 8 software (GraphPad Software, La Jolla, CA, USA) was used. Based on Shapiro–Wilk test, almost all data were skewed, so nonparametric tests were used. Significant differences between groups were assessed using the Mann–Whitney U test, and Kruskal–Wallis test was performed to compare data between all groups followed by Dunn’s post hoc test. Correlations were determined using Spearman’s ρ correlation. All tests were two-sided and significance was determined at *p* < 0.05. The analysis of microarray data by Z-score transformation was used in order to allow the comparison of microarray data independent of the original hybridization intensities [81]. Raw intensity data for each experiment is log10 transformed and then used for the calculation of Z scores. Z scores are calculated by subtracting the overall average gene intensity (within a single experiment) from the raw intensity data for each gene, and dividing that result by the SD of all of the measured intensities, according to the formula:

*Z score (intensity G—mean intensity G1. Gn)/SDG1. Gn* (1)

where G is any gene on the microarray and G1. Gn represent the aggregate measure of all of the genes [82].

The GSEA were expressed in weighted percentage and FDR as log10(2^-FDR) and graphically rendered in a circular diagram format using freely available CIRCOS software (Canada’s Michael Smith Genome Sciences Centre, Vancouver, Canada) [83]. CIRCOS can be applied to the exploration of data sets involving complex relationships between large numbers of factors.

Diagnostic accuracies were tested in logistic regression models separately for PMF versus healthy and PMF mutated versus PMF wild type. All models were evaluated for significance of the included biomarkers, overall diagnostic accuracy (area under the receiver operator characteristics curve, AUC), and overall fit penalized for the number of predictors (Akaike information criterion, AIC). Differences between AUCs were calculated in a bootstrap procedure with resampling (B = 1000 iterations).

## 5. Conclusions

Circulating CD34+ cells have a differential gene expression signature in PMF patients carrying the *JAK2^V617F^ mutation*, involving a dysregulation in immunoproteasome and *class I HLA* genes that could affect the interactions between neoplastic cells and the microenvironment. Further in-depth analysis looking at the type of antigens carried by the MHCs class I in the circulating CD34+ cells could disclose how the involvement of the IPs pathways can affect the clinical outcome of PMF.

## Figures and Tables

**Table 1:** Dataset information.

N°	Dataset	GPL	Healthy	PMF	CD34^+^	JAK2^V617F +^JAK2^V617F −^	
1	GSE53482	GPL13667	16	42	PB	23	19
2	GSE41812	GPL13667	0	20	PB	11	9

## Supplementary Materials

Supplementary materials can be found at https://www.mdpi.com/1422-0067/21/8/2926/s1.

## Abbreviations

c-20S	Proteasomes
MM	multiple myeloma
IPs	Immunoproteasome
TNFα	tumor necrosis factor alpha
IFNG	interferon gamma
LMP2 or PSMB9	subunits β1_i_
MECL-1 or PSMB10	subunits β2_i_
GEO	Gene Expression Omnibus
GHATER	Gene Annotation Tool to Help Explain Relationships
SDEG	Significantly Different Expressed Genes
MeV	MultiExperiment Viewer
MHC class I or HLA class I	Major Histocompatibility Complex class I
Th	helper T cell
TAP1 and TAP2	Transporter associated with Antigen Processing 1 and 2
PB	Peripheral Blood
