# Supplementary figures and images for: Immunoproteasome Genes Are Modulated in CD34+ JAK2V617F Mutated Cells from Primary Myelofibrosis Patients
Source: Int J Mol Sci. 2020 Apr 22;21(8):2926. doi: 10.3390/ijms21082926 (PMC7216198; doi:10.3390/ijms21082926)

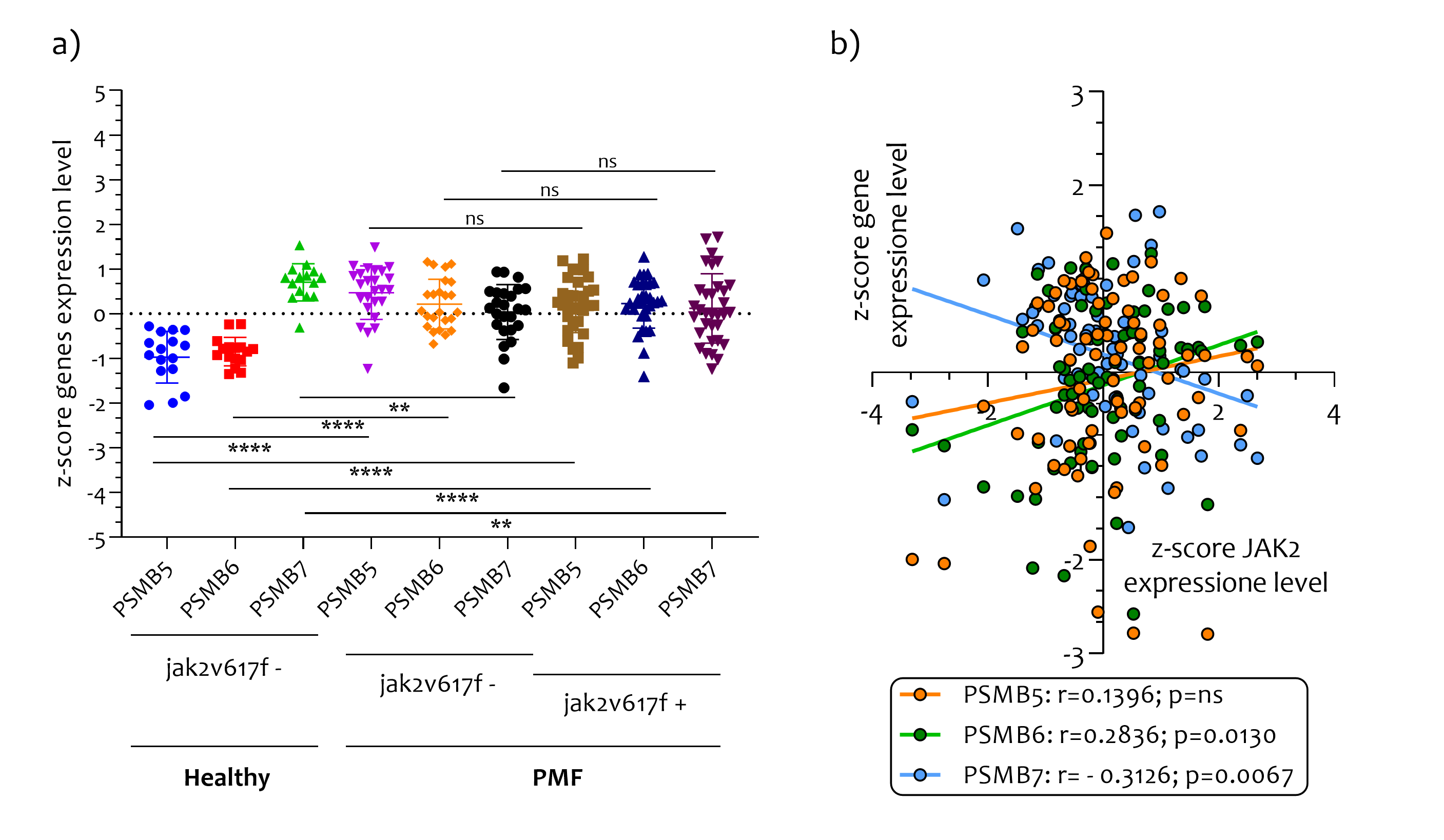

Supplement: Supplementary file 1 [file ijms-21-02926-s001.zip › supplementary files/Figure S1.tif]

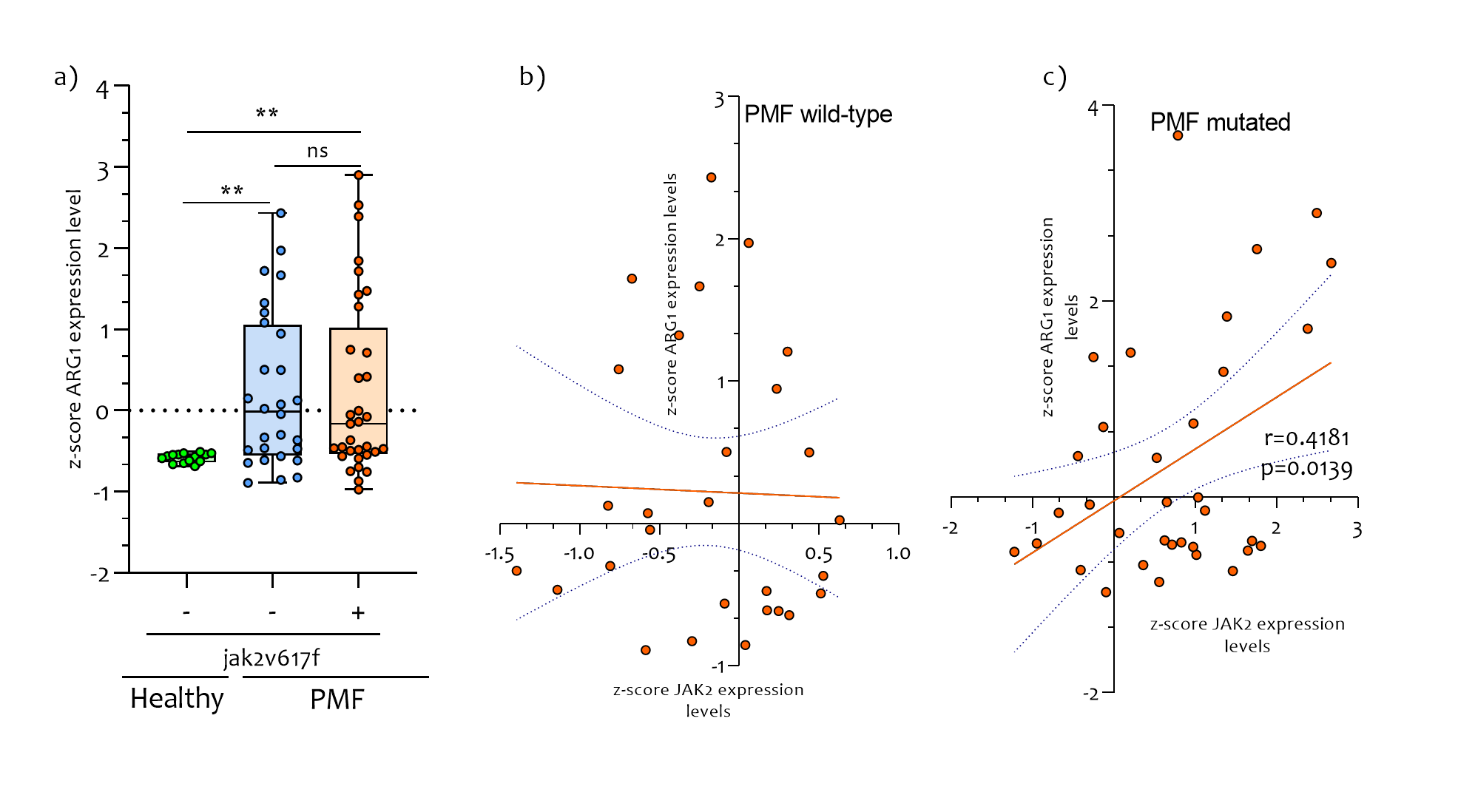

Supplement: Supplementary file 1 [file ijms-21-02926-s001.zip › supplementary files/Figure S2.tif]
